# Robust Optimization of Alginate-Carbopol 940 Bead Formulations

**DOI:** 10.1100/2012/605610

**Published:** 2012-05-01

**Authors:** J. M. López-Cacho, Pedro L. González-R, B. Talero, A. M. Rabasco, M. L. González-Rodríguez

**Affiliations:** ^1^Department of Pharmaceutical Technology, Faculty of Pharmacy, University of Seville, C/ Professor García González 2, 41012 Seville, Spain; ^2^Department of Industrial Management, School of Engineering, University of Seville, C/ Camino de los Descubrimientos s/n, 41092 Seville, Spain

## Abstract

Formulation process is a very complex activity which sometimes implicates taking decisions about parameters or variables to obtain the best results in a high variability or uncertainty context. Therefore, robust optimization tools can be very useful for obtaining high quality formulations. This paper proposes the optimization of different responses through the robust Taguchi method. Each response was evaluated like a noise variable, allowing the application of Taguchi techniques to obtain a response under the point of view of the signal to noise ratio. A *L*_18_ Taguchi orthogonal array design was employed to investigate the effect of eight independent variables involved in the formulation of alginate-Carbopol beads. Responses evaluated were related to drug release profile from beads (*t*_50%_ and AUC), swelling performance, encapsulation efficiency, shape and size parameters. Confirmation tests to verify the prediction model were carried out and the obtained results were very similar to those predicted in every profile. Results reveal that the robust optimization is a very useful approach that allows greater precision and accuracy to the desired value.

## 1. Introduction

The use of natural polymers for the design of drug delivery systems has long been the subject of great interest during the past decades. Sodium alginate is a sodium salt of alginic acid, a naturally occurring nontoxic polysaccharide found in marine brown algae. Alginate has been widely used as food and pharmaceutical additives, such as tablet disintegrant, thickening, and suspending agent. It contains two uronic acids, **α**-L-guluronic (G) and **β**-D-mannuronic acids (M), and is composed of homopolymeric blocks and blocks with an alternating sequence [1]. This polymer can form a reticulated structure when it contacts with Ca^2+^ and thus it has been used to produce sustained release particulate systems for various drugs, proteins, and even cells [2–4]. Gelation occurs by an ionic interaction between the calcium ions and the carboxylate anions of G-G blocks as calcium ions diffuse from the external source into the droplet forming a polyanionic microcapsule.

The addition of a polycation (poly-L-lysine or chitosan) to the gelation medium induces the formation of polyanionic-polycationic complexes, which stabilizes the ionic gel network and reduces the alginate permeability [5, 6].

The main advantage of using alginate to encapsulate drugs is that the alginate gelation process occurs under very mild conditions without using high temperatures or chemical crosslinking agents. Another advantage of using alginate is that the alginate gel can also be converted to sol by adding chelating agents, such as Na^+^ and EDTA. However, the drug releasing properties of Ca-sodium alginate matrices suffer from some serious problems. Firstly, the drugs could be leaked during the gel formation due to the long immersion time, which decreased the encapsulation efficiency. Secondly, the burst release of the drugs from pure Ca-sodium alginate beads is severe due to the quick breakdown of beads in the *in vitro* release process. Currently, much effort has been made for improving the performance of Ca-sodium alginate beads as drug delivery carriers.

Therefore, many factors are involved in the formulation of alginate microspheres. Some of them are summarized in Table 1.

Drug release from calcium-alginate beads depends on the swelling of the beads and the diffusion of the drug in the gel matrix [17]. Although alginate beads do not swell appreciably in acidic fluid [18], the beads swell and erode/disintegrate rapidly in the intestinal fluid, leading to a quick release of the loaded drug within a few minutes [6, 19] and hence calcium alginate matrix alone does not seem suitable as an oral controlled release system [20].

Polymeric materials have been widely used in order to conveniently modify and modulate the drug release from controlled-release microparticles. However, a large number of factors, including the chemical-physical properties of the raw materials (both drug and excipients), the composition and the relative amounts of the components in the formulations, as well as the manufacturing process parameters, can influence the drug release behavior from the final products [6, 7, 21, 22].

In the present study, we attempted to reinforce calcium-alginate beads containing methylene blue as a model drug by incorporating Carbopol 940 as hydrophilic polymer. This is a poly acrylic acid in anionic form that contains many free hydroxyl groups. Since it can form a gel, it has been used in combination with other polymers to control the drug release.

There are previous reports about the effect of carbomers on characteristics of calcium-alginate beads [23, 24] but not for Carbopol 940. Therefore, we intended to investigate the effect of this hydrophilic and swelling polymer on release behavior of calcium-alginate beads.

The preparation of calcium-alginate beads for drug entrapment is a stepwise procedure. The industrial experimentation includes a great number of parameters and a full factorial design that yield large number of experiments. Conventional optimization procedures involve altering one independent factor at a time keeping all others remain constant, which enables to assess the impact of those particular parameters on the process performance. These procedures are time consuming and cumbersome because require more experiments and cannot provide information about the mutual interactions of the parameters [25]. To reduce these numbers of experiments, a small set of trials with all possible combinations is selected by using various statistical tools [26].

Statistical experimental design methods, such as the fractional factorial design [8], the Plackett-Burman method [27], response surface methodology [28], the Box-Behnken design [29, 30], and the Taguchi method [31], provide a systematic and efficient plan for experimentation to achieve certain goals so that many control factors can be simultaneously studied. In contrast to the traditional “one-factor at a time” approach, these methods can be used to examine and optimize the operational variables while considering the interactive effects among the control factors.

The Taguchi method focuses on product robustness against uncontrollable influences, or noise. The method is designed to reduce variability, and optimize function or performance with the lowest cost components. Taguchi method of orthogonal array design of experiment (DOE) involves the study of any given system by a set of independent variables (factors) over a specific region of interest (levels). This approach also facilitates to identify the influence of individual factors, and establish the relationship between variables and operational conditions. In this methodology, the desired design is sought by selecting the best performance under conditions that produce consistent performance [32] and the results from small-scale experiments are valid over the scale up schedule [33].

Robustness is essential in the production of nearly all products. The variation in the quality of the product may follow from environmental factors and/or manufacturing variables that cannot be easily controlled (noise factors). In addition to emphasizing that the variability in the quality of the product and factors interactions should be reduced [34], the Taguchi method uses the signal-to-noise ratio (*S*/*N*), which is directly transformed from the quadratic quality loss function, as a measure to determine the robustness of a process. Thus, optimizing process parameters by the Taguchi method is an attempt not only to bring the average quality near to the target value, but also to simultaneously minimize the variation in quality [35]. Therefore, this method has been extensively applied in industry and its application caused significant changes in several industries, in manufacturing processes and total quality control [36–38].

This technique is somehow not very well known for optimization of pharmaceutical processes. This paper offers a rapid and efficient methodology to study and optimize pharmaceutical formulations, based on the Taguchi orthogonal array design. To do this, as an example, we used alginate-Carbopol beads formulated by ionotropic gelation with methylene blue (MB) as hydrophilic drug model.

After the selection of noise and control factors, we selected the profiles (responses) to optimize with the aim to demonstrate the applicability of this methodology in the optimization of different profiles such as drug release, swelling rate, or the morphology of the beads. Afterward, the experimental design and data analysis procedure, we selected the best profiles for Taguchi optimization and then we compared the results with predicted ones by lineal regression. Finally we compared the accuracy of the optimization.

## 2. Materials and Methods

### 2.1. Materials

Sodium alginate (viscosity 2% w/v: 250 cP) and triethanolamine (TEA) were purchased from Sigma Aldrich. Calcium chloride (Panreac, Spain) was selected as cation donor salt. MB was used as hydrophilic drug model. Carbopol 940 (Acofarma, Spain) was added to control the drug release from the alginate beads. Sodium tripolyphosphate and Tris(hydroxymethyl) aminomethane (TRIS) were obtained from Sigma (Spain).

### 2.2. Preparation of Beads

The beads were prepared by following the extrusion/precipitation method [39, 40]. Briefly, a sodium alginate aqueous solution containing 0.01% (w/v) MB and 2% (w/v) of sodium alginate was prepared by a simple mixing step. Carbopol 940 was dispersed in purified water and then was added to the above solution. As a function of the experiment, TEA was added to this solution. The beads were prepared by dropping the alginate-Carbopol solution (50 mL) containing MB from a syringe using a perfusion pump (Perfusor fm, Braun), through a 0.9 mm diameter needle into a gently stirred 0.25 M calcium chloride aqueous solution (IKA Eurostar). Different concentrations of Carbopol, at different dropping rate and stirring rates were used, as was reported in Table 2. The obtained hydrogels were maintained into the calcium chloride solution for different time periods to complete the chemical reaction. The beads were collected by decanting the calcium chloride solution, washed with deionized water and dried to a constant weight. At this stage, different drying methods were used: room temperature (20°C), rotary evaporator (reduced pressure, vacuum and 80°C), or oven (100°C).

### 2.3. Characterization of Beads

#### 2.3.1. Entrapment Efficiency

The MB content in the dry samples was determined by dissolving the beads, previously weighed, in a tripolyphosphate solution (0.03 M; 100 mL). The resulting solution was filtered and the drug concentration was analyzed by UV spectrophotometry, at 664 nm, using a UV-visible spectrophotometer (Hitachi U-2000). Entrapment efficiency was calculated as the percentage (w/w) of the theoretical drug content. Results were based on triplicate determinations.

#### 2.3.2. *In Vitro* Drug Release Studies

The drug release profiles of beads were determined using the USP 28 paddle method. The dissolution medium was 900 mL (sink conditions) of Tris-buffer solution adjusted at different pH and NaCl concentration values as a function of the experiment. The stirring speed was 50 rpm and the temperature was maintained at 37 ± 0.5°C. Accurately weighed samples of 700 mg beads were used for the test. At selected time intervals for a period of 420 min, aliquots each of 3 mL were withdrawn from de dissolution medium through a 0.45 *μ*m membrane filter and replaced with an equivalent amount of the fresh dissolution medium. Concentrations of MB were then spectrophotometrically determined at 664 nm. Each experiment was carried out in triplicate and the results were averaged.

Tris-buffer solution was selected as the dissolution medium because in the formation of alginate gel, alginate reaction with calcium ions turns reversible with sodium ions, and a disaggregation of the beads occurs [6]. We selected this buffer to control swelling process by modifying NaCl concentration.

#### 2.3.3. Swelling Process

The amount of absorbed water was determined following described methods by many authors [7, 41, 42]. In a first stage, a certain number of beads, accurately weighed (*W*_0_), were placed in glass beakers containing 80 mL of tris-buffer and NaCl concentration set by DOE. Next, the glass beakers were placed in a thermostatic bath at 25°C and 150 rpm. At time intervals of 1 h, beads were filtered and weighed (*W*_*e*_).

Swelling percentage (*E*_sw_) was calculated using the following expression [43]:

(2) $$E_{\text{SW}}=\left[\frac{W_{e}-W_{0}}{W_{0}}\right]\times 100,$$

where *W*_e_ is the wet weight of beads and *W*_0_ is the initial weight of beads.

#### 2.3.4. Morphological Properties of Beads

Morphological characteristics of beads were observed by Scanning Electron Microscopy, SEM (Philips XL30). The samples were sputter-coated with Au/Pd using a vacuum evaporator (Edwards) and examined at 20 kV accelerating voltage.

The size and shape of the beads were determined using image analyzer software (Image Pro Plus). The length and breadth were measured from the digital images of each bead and its size calculated from the average of these two dimensions [44].

For each formulation, 30 beads were randomly selected for measurement; the results were then averaged.

### 2.4. Optimization Methodology

**Figure 1:**
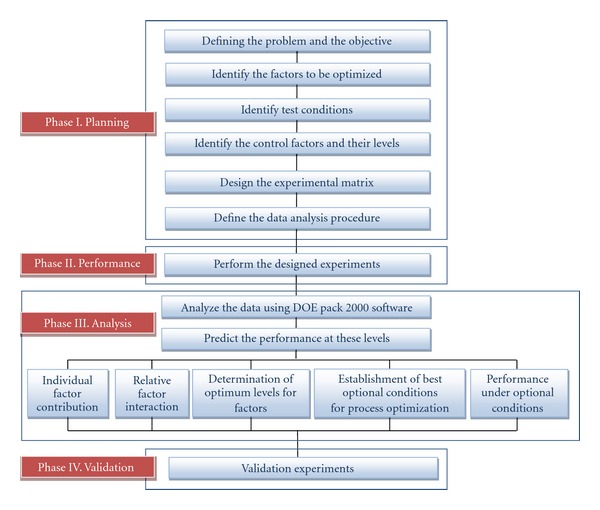
Schematic diagram of the steps involved in the Taguchi methodology.

Optimization methodology adopted in this study was divided into four phases (with various steps): planning, performance, analysis, and validation. The schematic representation of the designed methodology was depicted in Figure 1 (modified from [34]).

#### 2.4.1. Phase I. Planning

**(1) Defining the Problem and the Goal**

First step in Phase I is to determine the factors to be optimized in the production and characterization of alginate-Carbopol beads that have critical effect on the drug release, encapsulation efficiency, and in the morphological properties of the beads. The goal is to evaluate the effect of different factors on the elaboration of the beads and to obtain the perfect combination between the levels of the factors to optimize theirs properties.

**(2) Identifying the Factors to Be Optimized**

Six process parameters (flux rate, stirring rate, incubation time, drying mechanism, NaCl concentration, and pH of the dissolution medium) and two formulation parameters (presence of TEA and Carbopol concentration) were evaluated. The normal practice is to experiment with the feasible range, so that the variation inherent in the process does not mask the factors effects. The controls factor and theirs levels are exposed in Table 2.

**(3) Identifying the Test Conditions to Optimize**

The responses to be optimized from the characterization of alginate-Carbopol beads are those that have critical effect on the drug release, encapsulation efficiency, and morphological properties of the beads. Selected responses are exposed in Table 3.

**(4) Experimental Design and Data Analysis Procedure**

The fundamental principle of the Taguchi method is to improve the quality of a product by minimizing the effect of the causes of variation without eliminating the causes [45]. Three major tools used in the Taguchi method are the orthogonal arrays, analysis of variance (ANOVA), and the signal to noise ratio (*S*/*N*).

The Taguchi approach provides an opportunity to select a suitable orthogonal array depending on the number of control factors and theirs levels [46]. The orthogonal arrays give equal credence to all the parameters being investigated while ANOVA determines the contribution of each parameter in the design of the experiment. This is a fractional factorial approach and reduces the number of experiments.

Orthogonal array is a matrix of numbers arranged in rows and columns. Each row represents the level of factors in each run and each column represents a specific level for a factor that can be changed for each run.

*S*/*N* ratio is an indicative of quality, and the purpose of the Taguchi experiment is to find the best level for each operating parameter to maximize the ratio [47]. Analysis of the experimental data using the ANOVA and effects of the factors gives the output that is statistically significant for finding the optimum levels.

In the Taguchi method, quality is measured by the deviation of a characteristic from its target value, and a loss function [*L*(*y*)] is estimated for the deviation as *L*(*y*) = *k* × (*y*  −  *m*)^2^, where *k* denotes the proportionality constant, *m* represents the target value and *y* is the experimental value obtained for each trail. In case of bigger and better quality characteristics, the loss function can be written as *L*(*y*) = *k* × (1/*y* ^2^) and the expected loss function can be represented by:

(3) $$E\left[L\left(y\right)\right]=k\cdot E\left(\frac{1}{y^{2}}\right),$$

where *E*(1/*y* ^2^) can be estimated from *n* number of samples as:

(4) $$\frac{\sum_{i=1}^{n}\left[1/y_{i}^{2}\right]}{n}.$$

In order to measure the quadratic loss function, the Taguchi introduces the signal-to-noise (*S*/*N*) ratio for measuring the quality through orthogonal array based experiments. *S*/*N* ratios take into account both the variability in the response data and the closeness of the average response to a target value. The *S*/*N* ratio characteristics can be divided into three categories when the variable is continuous [48]:

(i) *nominal the best:*
  (5) $$\frac{S}{N}=-10\log\left(\frac{{\overset{®}{y}}_{i}^{2}}{\nu_{y_{n}}}\right),$$

(ii) *smaller the better:*
  (6) $$\frac{S}{N}=-10\log\left(\frac{\sum_{i}y_{i}^{2}}{n}\right),$$
(iii) *bigger the better:*
  $$\;\frac{S}{N}=-10\log\left(\frac{1/\sum_{i}y_{i}^{2}}{n}\right),$$

where $\overset{®}{y}$ is the average of observed data, *n* is the number of observations, and *y*_*i*_ the observed data for the *i*th run. In the nominal best study, *y* and *n* are nominal values those are to be achieved.

For each type of characteristics, with the above *S*/*N* ratio transformation, the higher the *S*/*N* ratio, the better is the result. The uncontrollable factors which cause the functional characteristics of a product to deviate from their target values are called noise factors. The overall aim of quality is to make products that are robust with respect to all noise factors [49].

The design was performed by an *L*_18_ array. In the present study, three levels of seven factors and two levels of one factor (Table 2) were considered and the size of experimentation was represented by symbolic array of *L*_18_ (which indicated 18 experimental trials) (Table 2). The three levels of all the factors have been assigned (except TEA (2^1^)), with a layout of *L*_18_  (2_1_ × 3^5^).

#### 2.4.2. Phase II. Performance of Experiments

After phase I, in which the factors to be optimized, theirs levels and the experimental matrix were selected, the experiments were carried out by triplicate (Table 4) and the selected variables were determined.

#### 2.4.3. Phase III. Analysis of the Data and Optimization

**(1) Analysis of Data**

At this stage, a statistical analysis of variance was performed to determine which test parameters were statistically significant for every variable. Moreover, the influence of each factor on the different response variables selected was determined.

**(2) Optimization**

After the analysis of the data, we proceeded to determine the optimal set parameter configuration. Optimization process was made applying the marginal means methodology (also called average rate or main effects analysis).

In order to do that, we obtained the average value of *S*/*N* ratio of every variable in every level of response and then the results were plotted in a graphic of marginal means. In the Taguchi method, we always tried to maximize the *S*/*N* ratio, so we select the levels with the higher values of *S*/*N* ratio in every factor that give us the maximum *S*/*N* final ratio. The difference in every response is the method followed for the consecution of the *S*/*N* ratio, as it was explained before. *S*/*N* ratios for every variable were obtained as Table 3 exposes. 

#### 2.4.4. Phase IV. Validation Experiments

With *S*/*N* ratio and ANOVA analysis, the optimal combination of the testing parameters could be predicted for a 95% confidence level. Also we can predict the future results that every combination is going to obtain by regression of the main influencing factors according to the ANOVA. 

In order to validate this methodology, validation experiments were further performed using the optimized conditions obtained for each response and then the results will be compared to the predicted by regression. If final results are similar to the predicted, we could validate the proposed methodology.

## 3. Results and Discussion

### 3.1. ANOVA

The application of the experimental design methodology has the objective of identifying the most significant factors influencing the formulation of alginate-Carbopol beads containing a water-soluble drug, and establishing the best levels for optimizing different response variables. 

Results obtained for these parameters, expressed as mean values, are presented in Table 4. ANOVA could be used as a tool for the detection of main effects. Data reported in Table 5 revealed that the release parameters (i.e., *t*_50%_ and AUC of release profiles) were significantly affected by Carbopol concentration and addition of TEA into the polymeric solution. Also, the presence of TEA significantly influenced on the entrapment efficiency and the particle size, whereas the swelling percentage was also affected by the NaCl concentration into the dissolution medium. Finally, the morphological parameters, expressed as shape factor, were mainly affected by the drying procedure and the stirring rate applied from the stirrer apparatus.

**Figure 2:**
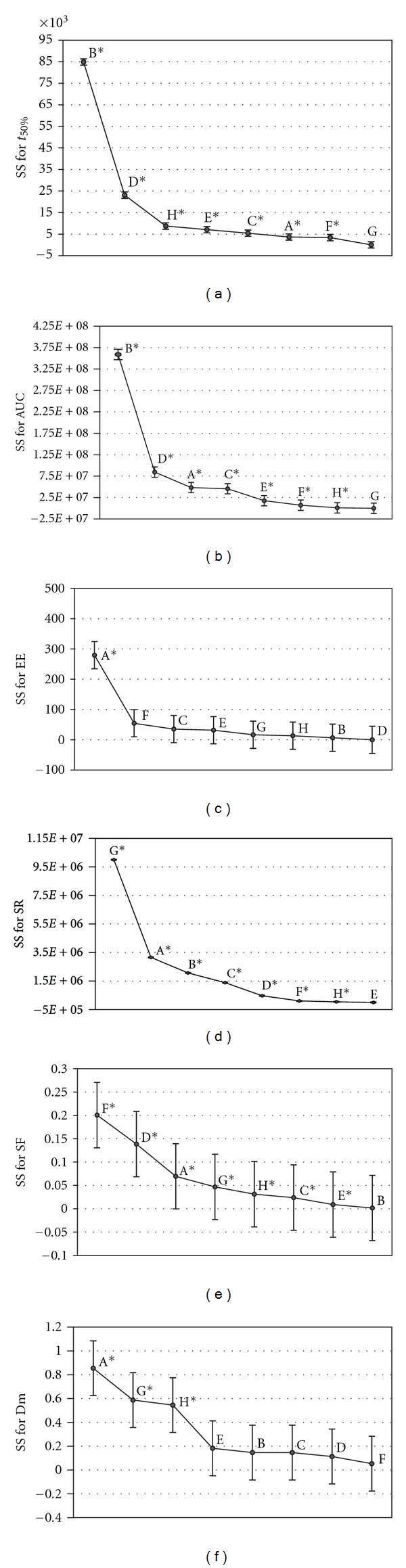
Scree plot obtained for the variables using the results of the ANOVA analysis. (a) Time to release 50% of entrapment drug, (b) area under the curve, (c) entrapment efficiency, (d) swelling rate, (e) shape factor, and (f) Diameter medium. *Values with statistical significance (*α* < 0,01). A: TEA; B: Carbopol (% w/v); C: flux rate (mL/min); D: stirring rate (rpm); E: retention time (min.); F: drying method; G: NaCl (% w/v); H: pH.

Contribution degrees of each factor to every response have been graphically showed in Figure 2. These findings will be discussed in next sections. 

#### 3.1.1. Release Profiles: Time to Release 50% of Entrapment Drug (*t*_50%_) and Area under the Curve (AUC)

As shown in Table 5, all factors except the NaCl concentration exert significant influence on *t*_50%_. In the case of AUC, NaCl concentration neither pH had influence on this response. From Figures 2(a) and 2(b), we can conclude that the Carbopol concentration (factor B) had the most significant effect on release characteristics. However, this effect is positive in case of *t*_50%_ (97.11) and negative in case of AUC (−6316). 

The influence of the Carbopol percentage on drug release profile is due to the effect of this polymer on the structure of the beads, as was discussed by other authors [50]. The density of the beads increases as the concentration of this polymer increases, suggesting that the beads formed at higher concentrations are more compact and less porous than those prepared at low polymer concentration [7]. The presence of a higher network density in the structure has been demonstrated by other authors in the preparation of Carbopol and PVP microspheres [16, 50]. Moreover, taking into account the swelling properties of this hydrophilic polymer, it is confirmed that the presence of the gel layer thickness acts as a barrier for the penetration medium thereby retarding the diffusion of MB [7, 11]. 

All these contribute to produce a slowing in MB release rate (increasing the *t*_50%_), making difficult the output of the drug to the dissolution medium (diminishing the AUC).

#### 3.1.2. Entrapment Efficiency

According to ANOVA analysis, the factor with higher contribution and statistical significance on the entrapment capacity of the drug was the incorporation of TEA into formulations. 

TEA is a weak base that ionizes carboxylic groups in the chains of carbomers. As a result, the structure of the gel changes its conformation, going from a relaxed state in spiral form to a rigid form, which provides viscosity and consistency to the beads. This structural conformation increases the entrapment efficiency. The same results have been obtained by other authors when they added chitosan to the alginate matrix to prepare beads [5]. 

Once the base was added, the increase of entrapment efficiency is as consequence of the creation of a higher network density and the electrostatic interaction between the drug and the polymers [16]. At the end of the process, the resulting beads become harder and thicker, favoring entrapment efficiency inside the beads but making difficult its later release. 

#### 3.1.3. Swelling Percentage

After the ANOVA analysis, we can determine that practically all factors have statistical influence on this dependent variable. The factor presenting the higher influence was NaCl concentration in the dissolution medium followed by TEA addition and Carbopol concentration. 

According to ANOM test for the factor NaCl concentration (data not showed), the presence of sodium ions acts hindering the swelling process. Swelling percentage will increase as NaCl concentration decreases (*I*-*hat* = −1293). 

**Figure 3:**
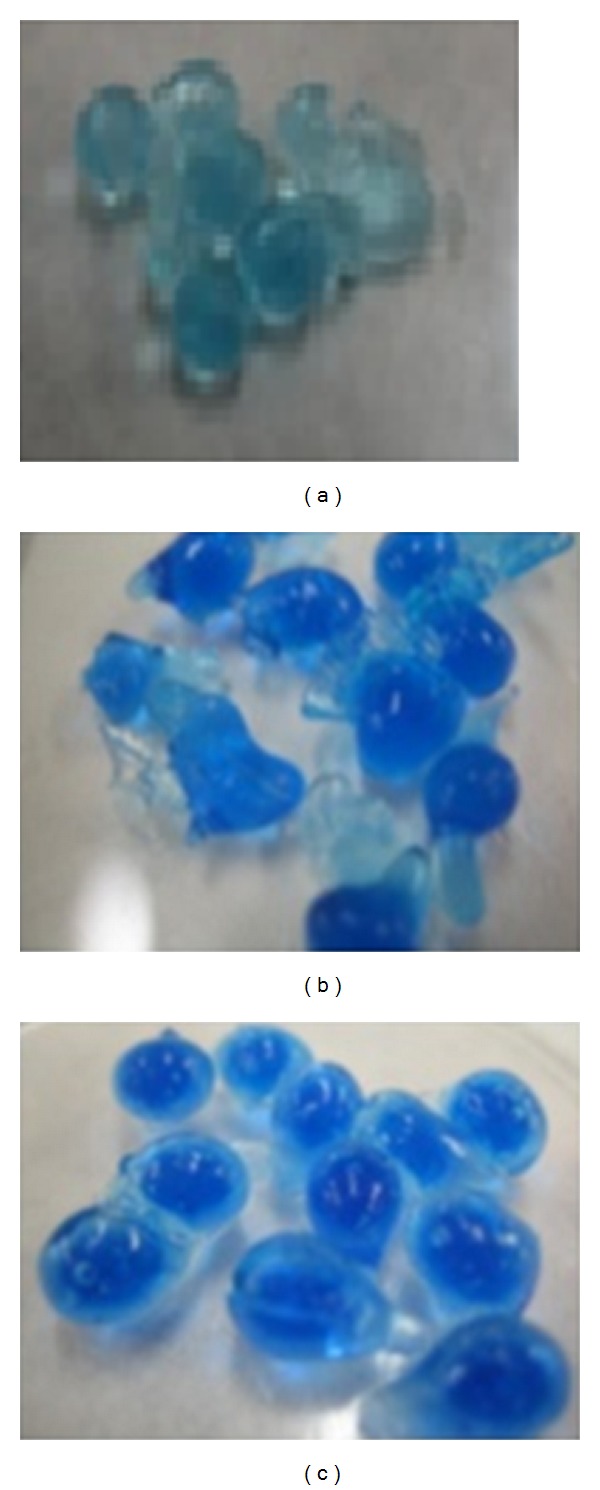
Digital images corresponding to samples during swelling tests (after 180 minutes). (a) batch 11 (0.1 M NaCl), (b) batch 15 (0.01 M NaCl) and (c) batch 16 (without NaCl).

This result can be explained on the basis of the swelling mechanism of these polymeric structures alginate-Carbopol. It is well accepted that the alginate beads in an aqueous medium without sodium ions do not swell well, but when these ions are present in the medium, the preformed complex between the alginate and calcium turns unstable [51, 52]. This could be explained by the ionotropic phenomenon that occurs between the Ca^2+^ ions of the alginate beads and the Na^+^ ions present in release medium that finally produces a capture of the Ca^2+^ by the chlorine ions. This fact produces a disaggregation of the bead structure leading to erosion and dissolution of the swollen beads and increasing the drug release rate [5, 9, 51]. This can be appreciated in Figure 3, where beads corresponding to batch 11 with higher NaCl concentration have lower swelling percentage at the same time than others. 

**Figure 4:**
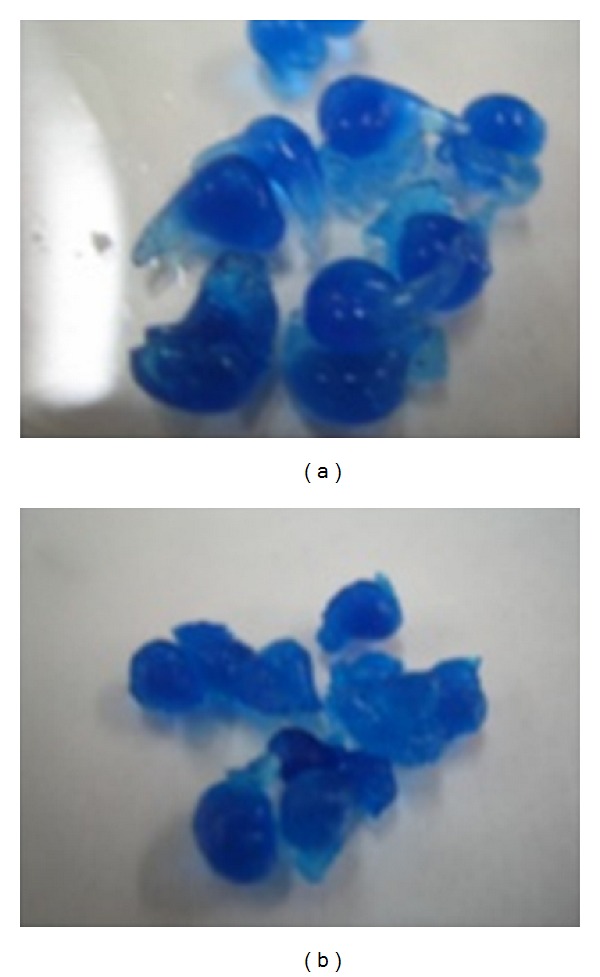
Digital images corresponding to samples during the swelling test. (a) Batch 17 (with TEA) and (b) batch 8 (without TEA).

In addition, TEA incorporation produces a rigid structure inside the beads and increase carboxylic groups with negative charge. These groups have a high affinity for water molecules so more quantity of water can get inside the bead, increasing the swelling of the beads in time and intensity. These results were visualized by photographing the samples during the swelling process tests. We can notice a higher swelling in batches with TEA addition (Figure 4) than in other lots without TEA at the same time. 

#### 3.1.4. Shape Factor

According to ANOVA analysis, the stirring rate and the drying method of beads are the factors that have the higher influence on this morphological parameter. 

**Figure 5:**
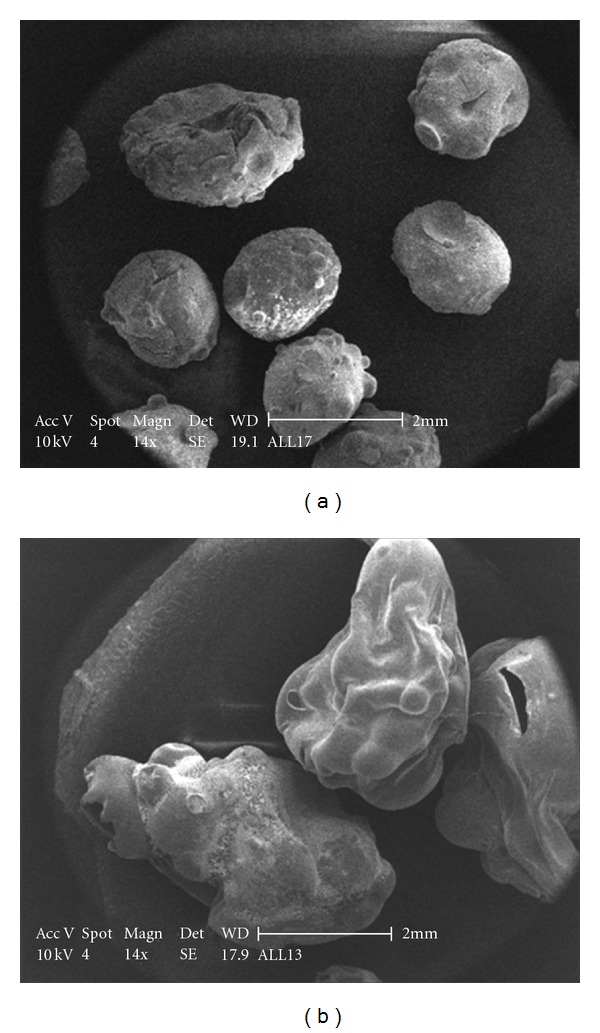
SEM images of alginate-Carbopol beads prepared with different stirring rates (a) 100 rpm (batch 11) and (b) 500 rpm (batch 14).

Stirring rate has an important statistical significance concerning the shape and size parameters [7]. When alginate-Carbopol drops fall into CaCl_2_ solution, stirring rate produce a force that molds the final morphological aspect of the beads, in relation to their shape and surface. As the stirring rate increases, beads will be less spherical and more irregular in its surface, which produces an increase of shape factor and a decrease in its circularity (inverse of SF), as we can demonstrate in Figure 5. 

From the evaluation of the drying process, we must keep in mind the characteristics of every treatment, because they can affect many properties of the beads [40]. Drying technique could influence several bead characteristics such as size [53–55], shape, mechanical properties [54], swelling properties [54, 55], drug release kinetics [56] and even drug migration to periphery of the beads along with water during drying [53, 57]. 

**Figure 6:**
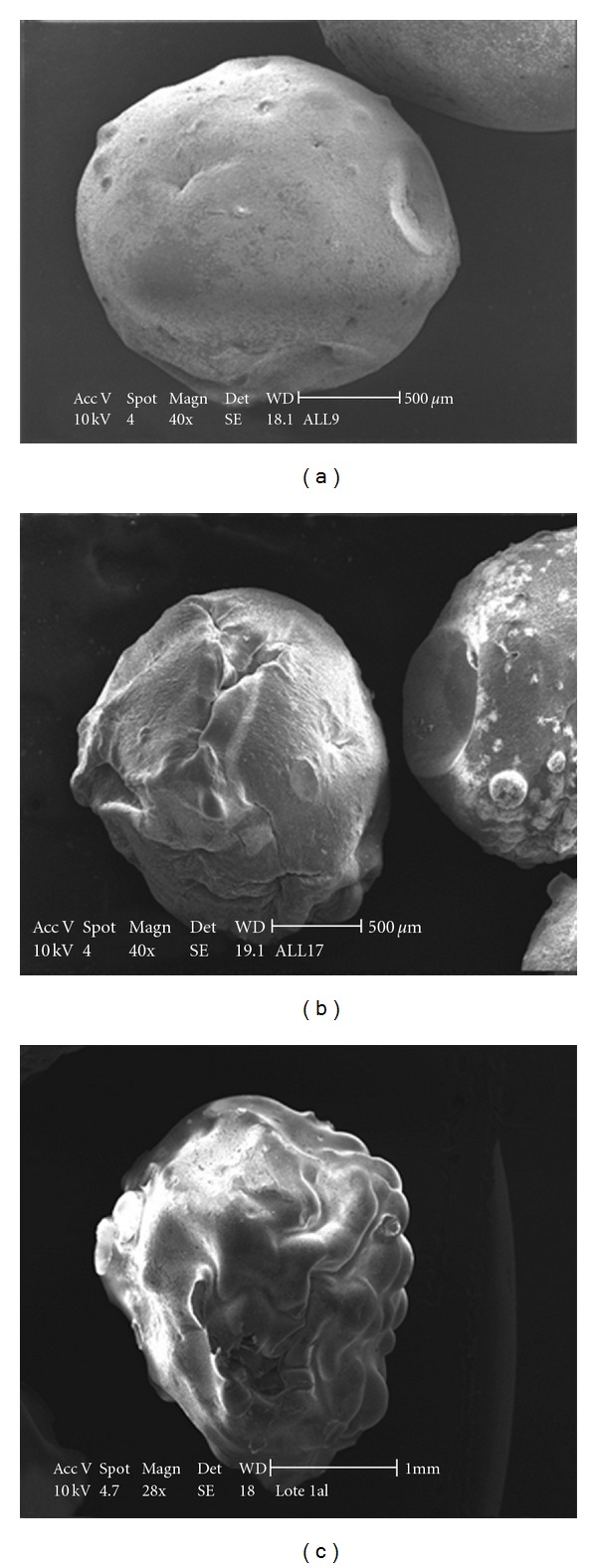
SEM images of beads dried during: (a) room temperature process (batch 8); (b) oven process (batch 11); (c) rotary evaporator process (batch 4).

When the beads were dried at environmental conditions, the water evaporation is slow and progressive. This slow loss of water allow the colloidal structure to settle slowly without getting to fracture point, obtaining at the end more spherical and smooth beads (Figure 6(a)). 

During the oven process, the drying temperature was about 100°C and hence, heat energy was supplied to the system, producing a strong and quick loss of water from the beads. As a consequence, the shape was altered producing roughs on the surface of the beads (Figure 6(b)). 

When the gelled structures were yielded in a rotary evaporator, the process was submitted to 80°C into a thermostatic bath and applying vacuum. In this case, the evaporation process is quicker than oven process even when we are applying less temperature. With the quicker loss of water, we obtain more irregular beads than oven process but with lesser imperfections on the surface of the beads (see Figure 6). 

Analyzing the effect produced by the different characteristics of each drying method, we can conclude that the irregular surface of the beads may be resulted mainly from the heat energy and the alteration on the shape of the beads is determined by the water loss rate. 

All these characteristics not only are going to influence on shape and size parameters, but also they are going to affect the release process [40]. As higher are size parameters we are going to obtain a quick release rate. In other words, the use of rotary evaporator as drying method will give rise to a quicker but less controlled drug release (low *t*_50%_ with high AUC).

#### 3.1.5. Diameter Medium

According to ANOVA test, TEA addition was the most significant contributor factor on particle size of the beads. As was explained above, the solutions containing TEA were more viscous. Then, when this polymer solution was dripped into CaCl_2_ solution, the gelled beads presented higher sizes and higher entrapment efficiencies [7]. 

### 3.2. Determination of Optimal Conditions Using Taguchi Robust Methodology

The appropriate selection of the parameters can improve the quality of the product, especially when at least two quality characteristics are to be simultaneously considered. The selection of the response parameters should account for the objectives of the study and the feasibility and ease of handling the optimization process [33]. 

**Figure 7:**
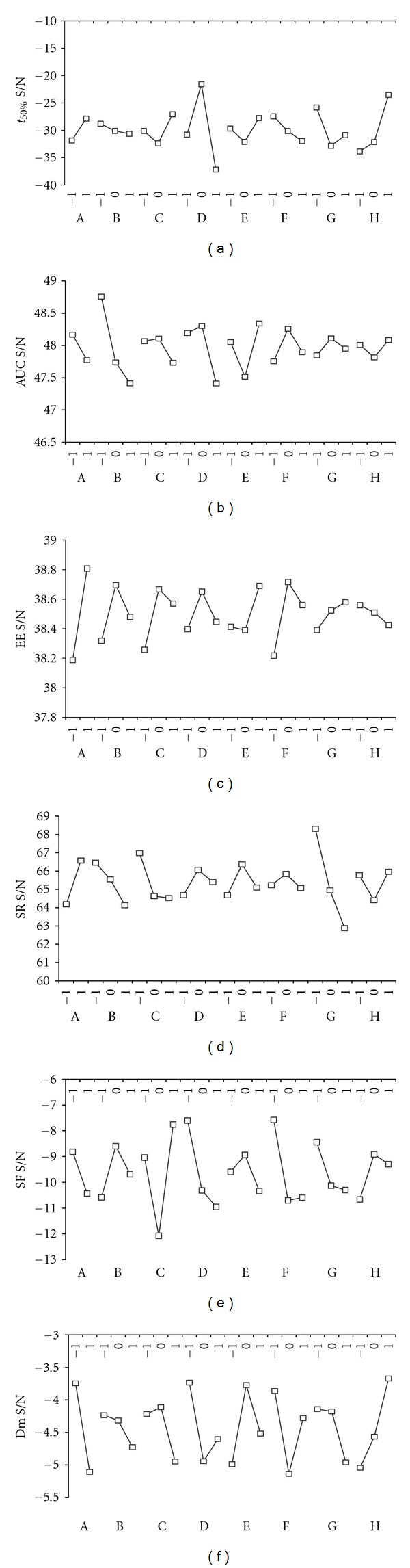
Marginal means graphs of the *S*/*N* ratio for (I) *t*_50%_, (II) AUC, (III) EE, (IV) SR, (V) SF, and (VI) Dm. (a): TEA, (b): Carbopol (% w/v), (c): Flux rate (mL/min), (d): Stirring rate (rpm), (e): Reaction time (min), (f): Drying method, (g): NaCl (% w/v), (h): pH.

The previous experiments revealed that the selected factors affected the release profile, the entrapment efficiency, the swelling process, and the morphological and dimensional properties of the beads depending to the analyzed variable. In the beginning of the optimization process, the marginal means graph for every response variable at each level of factors has been determined (Figure 7). Following these directives, we could select the best combination of factors and their levels that will give us the best results for each profile (Table 6). 

In order to validate this methodology, the confirmation tests were performed using their optimal combination of parameters levels. By using the predicted optimal combinations (Table 7) and a forecasting procedure (using linear regression) we can evaluate the adequacy of the optimizations if theirs results are closer to the predicted values.

For every evaluated response and following the combinations obtained by the Taguchi optimization and after confirmation tests by triplicate, we could see that predicted results are inside the confidence intervals obtained by practically all the optimal batches. 

The Taguchi optimization selects the best combination under conditions that produce consistent performance to obtain as possible an optimal value. In the Taguchi method, the robustness of a process is taken into more consideration than the final value of the optimized response. 

The success of the results by applying the Taguchi method is partly due to the ability to get good results in formulations or contexts with high variability. As argued in earlier sections, this variability is not always controllable by the researcher, so efficient methods to work in uncertain environments are always interesting to apply. 

Applying the Taguchi method to our results, we demonstrated that Taguchi method makes the final product less sensitive to variations than classical optimizations and that this methodology can be used as a tool for the optimization of other pharmaceutical forms.

## 4. Conclusions 

In the present work, we have focused on the applicability of the Taguchi robust methodology in the pharmaceutical field to optimize pharmaceutical processes. A technological process based on the production of alginate-Carbopol beads has been selected as an example, taking into account our experience in that field. 

Various formulation variables such stirring rate, flux rate, reaction time, drying method, and polymer concentration, and other variables such as NaCl concentration and pH were selected in the study. The influence of these parameters on drug entrapment efficiency, particle size, surface morphology, swelling behavior, and *in vitro* drug release has been analyzed. 

From the ANOVA tests, it may be concluded that the drug release rate from the beads was mainly affected by the Carbopol concentration and the stirring rate of the stirred during the bead formation process. The alginate-Carbopol MB-loaded beads had higher swelling percentages in absence of NaCl in the dissolution medium, and using higher polymer concentrations and with TEA into the polymeric solution. Beads dried at ambient conditions showed more satisfactory sustained release profile. 

The Taguchi design method was used to optimize the parameter values for obtaining the desired characteristics. Finally, the confirmation tests to verify the model prediction were carried out and the obtained values were very similar to the predicted ones. This result reveals the feasibility of robust optimization. Therefore, this methodology was then validated, being a perfect complement to the classical DOE methodology in environments with high variability and noise conditions.

## Figures and Tables

**Table 1:** Some of the factors affecting the encapsulation process of drugs into alginate beads.

Factor		Drug	Effect	Reference
Related with the formulation				
		Gliclazide		
Alginate concentration			+ EE	
			+ diameter	
			−porosity	[7]
			−release rate	
			+ swelling	
		BSA	+ EE	[5, 8]
		Glucose		

Volume of the alginate solution		Gliclazide	−diameter	[7]

Divalent cation concentration		BSA	−EE	[5, 8, 9]
		VEGF		
		Glucose		

Crosslinker/alginate ratio	Neutral (xanthan gums, ethylcellulose, PVA)	Diclofenac sodium	+ EE	[10, 11]
			+ swelling	
	Cationic (chitosan, poli-L-lysine)	Proteins Oligonucleotides	+ EE	[3, 5, 12]
	Waxy compounds (Compritol 888 ATO, Precirol ATO 5, Gelucires, glyceryl palmitostearate, magnesium aluminum silicate)	Tamsulosin, diclofenac sodium	+ EE −release rate	[13–16]
	Anionic (CMC, Eudragit L100, dextran sulphate) and cationic	Insulin	+EE	
			+ diameter	
			−release rate	

Surfactant presence		Gliclazide	−diameter	[7]
			+ EE	

Related with the process				
Needle size		BSA	−diameter	[5]
Coaxial air volume		BSA	−diameter	[5]
Drying process		BSA		[5]

Stirring rate		Gliclazide	−EE	[7]
			−diameter	

**Table 2:** Factors and their corresponding levels implemented for the construction of *L*_18_ orthogonal array.

Factor	Levels used, actual (coded)		
	Low (−1)	Medium (0)	High (+1)
Independent variables			
A: addition of TEA	No		Yes
B: concentration of Carbopol (% w/v)	1	2	4
C: flux rate of polymeric solution (mL/min)	1	2	3
D: stirring rate of stirrer (rpm)	100	300	500
E: reaction time of beads into CaCl_2_ solution (min)	5	30	60
F: drying method	Room	Rotary evaporator	Oven
G: NaCl (% w/v)	0	0.01	0.1
H: pH	7.2	7.6	8.0

**Table 3:** Test conditions to optimize.

Profile analyzed	Selected variable
Drug release	Time to release 50 percent of entrapment drug (*t*_50%_)
	Area under curve (AUC)
Entrapment efficiency	Entrapment efficiency (EE)
Swelling performance	Swelling rate (SR)
Shape of the particles	Shape factor (SF)
Size of the particles	Mean diameter (Dm)

**Table 4:** Experimental layout using a *L*_18_ orthogonal array and results obtained in every run.

Run		*t*_50%_		AUC		EE		SR		SF		Dm	
		Mean	*S*/*N*	Mean	*S*/*N*	Mean	*S*/*N*	Mean	*S*/*N*	Mean	*S*/*N*	Mean	*S*/*N*
1	*A*_−1_ *B*_−1_ *C*_−1_ *D*_−1_	73.00	28.82	268.68	49.11	48.43	37.04	2863.78	69.14	0.52	−5.74	1.34	−2.71
	*E*_−1_ *F*_−1_ *G*_−1_ *H*_−1_												
2	*A*_−1_ *B*_−1_ *C*_*o*_ *D*_*o*_	50.00	27.96	421.78	49.29	54.88	38.26	1937.37	65.74	0.20	−13.88	1.47	−3.43
	*E*_*o*_ *F*_*o*_ *G*_*o*_ *H*_0_												
3	*A*_−1_ *B*_−1_ *C*_1_ *D*_1_	69.33	35.57	117.23	48.60	53.63	38.08	1320.67	62.42	0.25	−12.06	1.53	−3.70
	*E*_1_ *F*_1_ *G*_1_ *H*_1_												
4	*A*_−1_ *B*_*o*_ *C*_−1_ *D*_−1_	91.33	31.94	461.89	48.23	54.88	38.17	1681.62	64.51	0.58	−4.77	1.72	−2.96
	*E*_*o*_ *F*_*o*_ *G*_1_ *H*_1_												
5	*A*_−1_ *B*_*o*_ *C*_*o*_ *D*_*o*_	102.33	32.18	405.74	48.66	59.00	38.92	2177.52	66.76	0.27	−11.32	1.64	−4.50
	*E*_1_ *F*_1_ *G*_−1_ *H*_−1_												
6	*A*_−1_ *B*_*o*_ *C*_1_ *D*_1_	198.33	25.60	348.62	47.04	53.51	38.08	1119.52	60.98	0.65	−3.83	1.69	−4.61
	*E*_−1_ *F*_−1_ *G*_*o*_ *H*_0_												
7	*A*_−1_ *B*_1_ *C*_−1_ *D*_*o*_	118.67	19.17	354.48	48.22	54.34	38.21	1610.31	64.14	0.30	−10.47	1.51	−3.65
	*E*_−1_ *F*_1_ *G*_*o*_ *H*_1_												
8	*A*_−1_ *B*_1_ *C*_*o*_ *D*_1_	293.33	28.10	257.57	46.46	54.46	38.24	1082.83	60.69	0.25	−12.01	1.54	−3.73
	*E*_*o*_ *F*_−1_ *G*_1_ *H*_−1_												
9	*A*_−1_ *B*_1_ *C*_1_ *D*_−1_	106.00	18.80	427.85	47.90	57.14	38.65	1459.06	63.28	0.55	−5.26	1.66	−4.42
	*E*_1_ *F*_*o*_ *G*_−1_ *H*_0_												
10	*A*_1_ *B*_−1_ *C*_−1_ *D*_1_	60.00	24.77	448.50	48.89	59.73	39.04	2786.18	68.90	0.19	−14.68	2.07	−6.33
	*E*_1_ *F*_*o*_ *G*_*o*_ *H*_−1_												
11	*A*_1_ *B*_−1_ *C*_*o*_ *D*_−1_	82.67	22.18	634.32	48.71	59.33	38.97	1151.14	61.22	0.26	−11.95	1.83	−5.27
	*E*_−1_ *F*_1_ *G*_1_ *H*_0_												
12	*A*_1_ *B*_−1_ *C*_1_ *D*_*o*_	119.33	40.29	401.70	47.93	56.07	38.49	3685.23	71.32	0.55	−5.20	1.57	−3.97
	*E*_*o*_ *F*_−1_ *G*_−1_ *H*_1_												
13	*A*_1_ *B*_*o*_ *C*_−1_ *D*_*o*_	111.67	23.30	1326.03	47.92	58.83	38.91	1868.79	65.39	0.40	−9.30	1.73	−5.12
	*E*_1_ *F*_−1_ *G*_1_ *H*_0_												
14	*A*_1_ *B*_*o*_ *C*_*o*_ *D*_1_	148.67	36.17	225.15	47.45	59.88	39.06	3003.21	69.55	0.20	−13.88	1.72	−4.70
	*E*_−1_ *F*_*o*_ *G*_−1_ *H*_1_												
15	*A*_1_ *B*_*o*_ *C*_1_ *D*_−1_	209.33	36.72	469.47	47.14	59.49	39.00	2017.31	66.10	0.38	−8.50	1.56	−4.01
	*E*_*o*_ *F*_1_ *G*_*o*_ *H*_−1_												
16	A_1_B_1_C_−1_D_1_	238.67	40.29	1052.68	46.04	53.97	38.15	3094.41	69.81	0.35	−9.26	1.67	−4.54
	*E*_*o*_ *F*_1_ *G*_−1_ *H*_0_												
17	*A*_1_ *B*_1_ *C*_*o*_ *D*_−1_	142.00	32.25	536.70	48.08	56.39	38.52	1554.57	63.83	0.34	−9.40	1.41	−3.04
	*E*_1_ *F*_−1_ *G*_*o*_ *H*_1_												
18	*A*_1_ *B*_1_ *C*_1_ *D*_*o*_	138.33	33.61	841.84	47.79	60.02	39.08	1420.16	63.05	0.26	−11.71	2.81	−8.99
	*E*_−1_ *F*_*o*_ *G*_1_ *H*_−1_												

**Table 5:** ANOVA results: effect of the variables on *t*_50%_, AUC, % entrapment (EE), swelling percentage (SR), shape factor (SF), and particle size (DM).

Factors	*t*_50%_			AUC			EE		
	*F*-ratio	*P*	*I*-*hat*	*F*-ratio	*P*	*I*-*hat*	*F*-ratio	*P*	*I*-*hat*
A	89.52	<0.001	16.48	144.1	<0.001	−1892	11.2	0.004	5.57
B	2072	<0.001	97.11	1070	<0.001	−6316	0.27	0.607	1.07
C	132.5	<0.001	24.56	136	<0.001	−2252	1.41	0.25	2.42
D	564	<0.001	50.67	251.7	<0.001	−3063	0.003	0.955	−0.12
E	172.9	<0.001	−28.06	52.87	<0.001	1404	1.27	0.274	2.30
F	83.07	<0.001	−19.44	21.36	<0.001	892.2	2.18	0.157	3.01
G	0.01	0.918	−0.22	0.01	0.921	−19.4	0.67	0.425	1.66
H	213.4	<0.001	−31.17	3.20	0.082	345.2	0.53	0.474	−1.49

Factors	SR			SF			DM		
	*F*-ratio	*P*	*I*-*hat*	F-ratio	*P*	*I*-*hat*	*F*-ratio	*P*	*I*-*hat*

A	1842	<0.001	592	34.79	<0.001	−0.072	12.78	<0.001	0.255
B	1208	<0.001	−587.2	0.78	0.383	0.013	2.19	0.148	0.128
C	808.8	<0.001	−480.5	11.9	<0.001	0.051	2.19	0.148	0.130
D	274.4	<0.001	279.9	69.35	<0.001	−0.124	1.69	0.201	0.115
E	0.00	0.99	−0.22	4.49	0.041	−0.032	2.73	0.108	−0.142
F	62.8	<0.001	−133.9	100.4	<0.001	−0.149	0.80	0.377	0.077
G	5856	<0.001	−1293	23.4	<0.001	−0.072	8.78	0.005	0.261
H	25.09	<0.001	84.64	15.63	<0.001	0.059	8.15	0.007	−0.251

**Table 6:** Optimization process carried out in each variable for robust method.

Variable	Aim in Taguchi	Robust
*t*_50%_	Smaller is best (to120 minutes)	*A*_1_ *B*_−1_ *C*_1_ *D*_0_ *E*_1_ *F*_−1_ *G*_−1_ *H*_1_
AUC	Bigger is best	*A*_−1_ *B*_−1_ *C*_0_ *D*_0_ *E*_1_ *F*_0_ *G*_0_ *H*_1_
EE	Bigger is best	*A*_1_ *B*_0_ *C*_0_ *D*_0_ *E*_1_ *F*_0_ *G*_1_ *H*_−1_
SR	Bigger is best	*A*_1_ *B*_−1_ *C*_−1_ *D*_0_ *E*_0_ *F*_0_ *G*_−1_ *H*_1_
SF	Bigger is best	*A*_−1_ *B*_0_ *C*_1_ *D*_−1_ *E*_0_ *F*_−1_ *G*_−1_ *H*_0_
Dm	Smaller is best	*A*_−1_ *B*_−1_ *C*_0_ *D*_−1_ *E*_0_ *F*_−1_ *G*_−1_ *H*_1_

**Table 7:** Results obtained by confirmation test. SD: standard deviation; CI: confidence interval.

Variable	Mean	Predicted	SD	CI (*α* = 0.05)
*t*_50%_	180	186	6.34	180 ± 7.17
AUC	19359.31	26086.38	613.57	24350.77 ± 694.3
EE	85.55	87.28	2.32	85.55 ± 2.62
SR	3073.33	3509.44	122.20	3073.33 ± 138.28
SF	0.44	0.47	0.10	0.44 ± 0.06
Dm	1.37	1.55	0.15	1.37 ± 0.16

## References

[1] Draget KI, Philips GO, Williams PA (2000). Alginates. *Handbook of Hydrocolloids*.

[2] Orive G, Ponce S, Hernández RM, Gascón AR, Igartua M, Pedraz JL (2002). Biocompatibility of microcapsules for cell immobilization elaborated with different type of alginates. *Biomaterials*.

[3] George M, Abraham TE (2006). Polyionic hydrocolloids for the intestinal delivery of protein drugs: alginate and chitosan—a review. *Journal of Controlled Release*.

[4] Kim YS, Kim HW, Lee SH, Shin KS, Hur HW, Rhee YH (2007). Preparation of alginate-quaternary ammonium complex beads and evaluation of their antimicrobial activity. *International Journal of Biological Macromolecules*.

[5] Vandenberg GW, Drolet C, Scott SL, de la Noüe J (2001). Factors affecting protein release from alginate-chitosan coacervate microcapsules during production and gastric/intestinal simulation. *Journal of Controlled Release*.

[6] González-Rodríguez ML, Holgado MA, Sánchez-Lafuente C, Rabasco AM, Fini A (2002). Alginate/chitosan particulate systems for sodium diclofenac release. *International Journal of Pharmaceutics*.

[7] Al-Kassas RS, Al-Gohary OMN, Al-Faadhel MM (2007). Controlling of systemic absorption of gliclazide through incorporation into alginate beads. *International Journal of Pharmaceutics*.

[8] McEntee MKE, Bhatia SK, Tao L, Roberts SC, Bhatia SR (2008). Tunable transport of glucose through ionically-crosslinked alginate gels: effect of alginate and calcium concentration. *Journal of Applied Polymer Science*.

[9] Gu F, Amsden B, Neufeld R (2004). Sustained delivery of vascular endothelial growth factor with alginate beads. *Journal of Controlled Release*.

[10] Pongjanyakul T, Puttipipatkhachorn S (2007). Xanthan-alginate composite gel beads: molecular interaction and in vitro characterization. *International Journal of Pharmaceutics*.

[11] Hua S, Ma H, Li X, Yang H, Wang A (2010). pH-sensitive sodium alginate/poly(vinyl alcohol) hydrogel beads prepared by combined Ca2+ crosslinking and freeze-thawing cycles for controlled release of diclofenac sodium. *International Journal of Biological Macromolecules*.

[12] González Ferreiro M, Tillman L, Hardee G, Bodmeier R (2002). Characterization of alginate/poly-L-lysine particles as antisense oligonucleotide carriers. *International Journal of Pharmaceutics*.

[13] Kim MS, Park GD, Jun SW, Lee S, Park JS, Hwang SJ (2005). Controlled release tamsulosin hydrochloride from alginate beads with waxy materials. *Journal of Pharmacy and Pharmacology*.

[14] Pongjanyakul T, Sungthongjeen S, Puttipipatkhachorn S (2006). Modulation of drug release from glyceryl palmitostearate-alginate beads via heat treatment. *International Journal of Pharmaceutics*.

[15] Puttipipatkhachorn S, Pongjanyakul T, Priprem A (2005). Molecular interaction in alginate beads reinforced with sodium starch glycolate or magnesium aluminum silicate, and their physical characteristics. *International Journal of Pharmaceutics*.

[16] Silva CM, Ribeiro AJ, Ferreira D, Veiga F (2006). Insulin encapsulation in reinforced alginate microspheres prepared by internal gelation. *European Journal of Pharmaceutical Sciences*.

[17] Sugawara S, Imai T, Otagiri M (1994). The controlled release of prednisolone using alginate gel. *Pharmaceutical Research*.

[18] Yotsuyanagi T, Ohkubo T, Ohhashi T, Ikeda K (1987). Calcium-induced gelation of alginic acid and pH-sensitive reswelling of dried gels. *Chemical and Pharmaceutical Bulletin*.

[19] Tomida H, Mizuo C, Nakamura C, Kiryu S (1993). Imipramine release from Ca-alginate gel beads. *Chemical and Pharmaceutical Bulletin*.

[20] Ostberg T, Lund EM, Graffner C (1994). Calcium alginate matrices for oral multiple unit administration IV. Release characteristics in different media. *International Journal of Pharmaceutics*.

[21] Mi FL, Sung HW, Shyu SS (2002). Drug release from chitosan-alginate complex beads reinforced by a naturally occurring cross-linking agent. *Carbohydrate Polymers*.

[22] Tapia C, Escobar Z, Costa E (2004). Comparative studies on polyelectrolyte complexes and mixtures of chitosan-alginate and chitosan-carrageenan as prolonged diltiazem clorhydrate release systems. *European Journal of Pharmaceutics and Biopharmaceutics*.

[23] Neau SH, Chow MY, Hileman GA, Durrani MJ, Gheyas F, Evans BA (2000). Formulation and process considerations for beads containing Carbopol^®^ 974P, NF resin made by extrusion-spheronization. *International Journal of Pharmaceutics*.

[24] Bommareddy GS, Paker-Leggs S, Saripella KK, Neau SH (2006). Extruded and spheronized beads containing Carbopol^®^ 974P to deliver nonelectrolytes and salts of weakly basic drugs. *International Journal of Pharmaceutics*.

[25] Subba Rao C, Madhavendra SS, Sreenivas Rao R, Hobbs PJ, Prakasham RS (2008). Studies on improving the immobilized bead reusability and alkaline protease production by isolated immobilized bacillus circulans (MTCC 6811) using overall evaluation criteria. *Applied Biochemistry and Biotechnology*.

[26] Saudagar PS, Singhal RS (2007). Optimization of nutritional requirements and feeding strategies for clavulanic acid production by Streptomyces clavuligerus. *Bioresource Technology*.

[27] Potumarthi R, Subhakar C, Pavani A, Jetty A (2008). Evaluation of various parameters of calcium-alginate immobilization method for enhanced alkaline protease production by *Bacillus licheniformis* NCIM-2042 using statistical methods. *Bioresource Technology*.

[28] Dagbagli S, Goksungur Y (2008). Optimization of *β*-galactosidase production using Kluyveromyces lactis NRRL Y-8279 by response surface methodology. *Electronic Journal of Biotechnology*.

[29] Singh N, Seedat F, Pillay V, Sweet JL, Danckwerts MP (2006). Formulation and statistical optimization of novel double-incorporated PLA-PLGA microparticles within an alginate-pectinate platform for the delivery of nicotine. *Journal of Microencapsulation*.

[30] Gazori T, Khoshayand MR, Azizi E, Yazdizade P, Nomani A, Haririan I (2009). Evaluation of Alginate/Chitosan nanoparticles as antisense delivery vector: formulation, optimization and in vitro characterization. *Carbohydrate Polymers*.

[31] Rao RS, Kumar CG, Prakasham RS, Hobbs PJ (2008). The Taguchi methodology as a statistical tool for biotechnological applications: a critical appraisal. *Biotechnology Journal*.

[32] Roy RK (2001). *Design of Experiments Using the Taguchi Approach: 16 Steps to Product and Process Improvement*.

[33] Houng JY, Hsu HF, Liu YH, Wu JY (2003). Applying the Taguchi robust design to the optimization of the asymmetric reduction of ethyl 4-chloro acetoacetate by bakers’ yeast. *Journal of Biotechnology*.

[34] Adnani A, Basri M, Malek EA (2010). Optimization of lipase-catalyzed synthesis of xylitol ester by Taguchi robust design method. *Industrial Crops and Products*.

[35] Taguchi G (1987). System of experimental design. *Engineering Methods to Optimize Quality and Minimize Costs*.

[36] Mandal V, Mohan Y, Hemalatha S (2008). Microwave assisted extraction of curcumin by sample-solvent dual heating mechanism using Taguchi L9 orthogonal design. *Journal of Pharmaceutical and Biomedical Analysis*.

[37] Lee SH, Heng D, Ng WK, Chan HK, Tan RBH (2011). Nano spray drying: a novel method for preparing protein nanoparticles for protein therapy. *International Journal of Pharmaceutics*.

[38] Tong LI, Wang CH, Chen CC, Chen CT (2004). Dynamic multiple responses by ideal solution analysis. *European Journal of Operational Research*.

[39] Cerdeira AM, Gouveia LF, Goucha P, Almeida AJ (2000). Drug particle size influence on enteric beads produced by a droplet extrusion/precipitation method. *Journal of Microencapsulation*.

[40] Smrdel P, Bogataj M, Mrhar A (2008). The influence of selected parameters on the size and shape of alginate beads prepared by ionotropic gelation. *Scientia Pharmaceutica*.

[41] Anal AK, Stevens WF (2005). Chitosan-alginate multilayer beads for controlled release of ampicillin. *International Journal of Pharmaceutics*.

[42] Basu SK, Rajendran A (2008). Studies in the development of nateglinide loaded calcium alginate and chitosan coated calcium alginate beads. *Chemical and Pharmaceutical Bulletin*.

[43] Roger S, Talbot D, Bee A (2006). Preparation and effect of Ca^2+^ on water solubility, particle release and swelling properties of magnetic alginate films. *Journal of Magnetism and Magnetic Materials*.

[44] Albertini B, Passerini N, González-Rodríguez ML, Perissutti B, Rodriguez L (2004). Effect of Aerosil^®^ on the properties of lipid controlled release microparticles. *Journal of Controlled Release*.

[45] Jahanshahi M, Sanati MH, Babaei Z (2008). Optimization of parameters for the fabrication of gelatin nanoparticles by the Taguchi robust design method. *Journal of Applied Statistics*.

[46] Mousavi SM, Yaghmaei S, Jafari A, Vossoughi M, Ghobadi Z (2007). Optimization of ferrous biooxidation rate in a packed bed bioreactor using Taguchi approach. *Chemical Engineering and Processing*.

[47] Khosla A, Kumar S, Aggarwal KK (2006). Identification of strategy parameters for particle swarm optimizer through Taguchi method. *Journal of Zhejiang University Science*.

[48] Tan O, Zaimoglu AS, Hinislioglu S, Altun S (2005). Taguchi approach for optimization of the bleeding on cement-based grouts. *Tunnelling and Underground Space Technology*.

[49] Sahin Y (2006). Optimal testing parameters on the wear behaviour of various steels. *Materials and Design*.

[50] Chun MK, Cho CS, Choi HK (2005). Mucoadhesive microspheres prepared by interpolymer complexation and solvent diffusion method. *International Journal of Pharmaceutics*.

[51] Thu B, Bruheim P, Espevik T, Smidsrød O, Soon-Shiong P, Skjåk-Bræk G (1996). Alginate polycation microcapsules: II. Some functional properties. *Biomaterials*.

[52] Türkoglu M, Gürsoy A, Eroglu L, Okar I (1997). Effect of aqueous polymer dispersions on properties of diclofenac/alginate beads and in vivo evaluation in rats. *S.T.P. Pharma Sciences*.

[53] Fundueanu G, Nastruzzi C, Carpov A, Desbrieres J, Rinaudo M (1999). Physico-chemical characterization of Ca-alginate microparticles produced with different methods. *Biomaterials*.

[54] Gal A, Nussinovitch A (2007). Hydrocolloid carriers with filler inclusion for diltiazem hydrochloride release. *Journal of Pharmaceutical Sciences*.

[55] George M, Abraham TE (2007). pH sensitive alginate-guar gum hydrogel for the controlled delivery of protein drugs. *International Journal of Pharmaceutics*.

[56] Sriamornsak P (1998). Investigation of pectin as a carrier for oral delivery of proteins using calcium pectinate gel beads. *International Journal of Pharmaceutics*.

[57] Shariff A, PK M, KLK P, M M (2007). Entrapment of andrographolide in cross-linked alginate pellets: I. Formulation and evaluation of associated release kinetics. *Pakistan Journal of Pharmaceutical Sciences*.

